# *In Vivo* Quantification of Vcam-1 Expression in Renal Ischemia Reperfusion Injury Using Non-Invasive Magnetic Resonance Molecular Imaging

**DOI:** 10.1371/journal.pone.0012800

**Published:** 2010-09-21

**Authors:** Asim M. Akhtar, Jurgen E. Schneider, Stephanie J. Chapman, Andrew Jefferson, Janet E. Digby, Kulveer Mankia, Ye Chen, Martina A. McAteer, Kathryn J. Wood, Robin P. Choudhury

**Affiliations:** 1 Department of Cardiovascular Medicine, University of Oxford, John Radcliffe Hospital, Oxford, United Kingdom; 2 Transplantation Research Immunology Group, Nuffield Department of Surgical Sciences, University of Oxford, John Radcliffe Hospital, Oxford, United Kingdom; University of Padova, Medical School, Italy

## Abstract

**Rationale and Objective:**

Vascular cell adhesion molecule-1 (VCAM-1) is upregulated in ischemia reperfusion injury (IRI), persisting after restoration of blood flow. We hypothesized that microparticles of iron oxide targeting VCAM-1 (VCAM-MPIO) would depict “ischemic memory” and enable *in vivo* assessment of VCAM-1 expression.

**Methodology and Findings:**

Mice subject to unilateral, transient (30 minutes) renal ischemia and subsequent reperfusion received intravenous VCAM-MPIO (4.5 mg iron/kg body weight). Contrast agent bound rapidly (<30 minutes) in IRI-kidneys and appeared as intensely low signal areas by MRI *in vivo*. Automated segmentation and quantification yielded MPIO contrast volumes of 5991±354×10^6^ µm^3^ in IRI *vs*. 87±7×10^6^ µm^3^ in kidneys with no surgical intervention (*P*<0.001); 90±8×10^6^ µm^3^ in IRI kidneys exposed to control (IgG-MPIO) and 625±80×10^6^ µm^3^, in IRI kidneys pre-treated with a blocking dose of VCAM-1 antibody (*P*<0.001). In keeping with quantitative MRI data, VCAM-1 mRNA expression in IRI was 65-fold higher than in kidneys without surgical intervention (3.06±0.63 *vs.* 0.05±0.02, P<0.001). Indeed VCAM-1 mRNA expression and VCAM-MPIO contrast volume were highly correlated (R^2^ = 0.901, P<0.01), indicating that quantification of contrast volume reflected renal VCAM-1 transcription. Serial imaging showed VCAM-MPIO accumulation at target within 30 minutes, persisting for ≥90 minutes, while unbound VCAM-MPIO was cleared rapidly from blood, with sequestration by mac-3 positive Kupffer cells in the liver and monocyte/macrophages in the spleen.

**Conclusions:**

(1) VCAM-MPIO detected VCAM-1 expression and defined its 3-dimensional distribution, revealing “ischemic memory” in renal IRI; (2) automated volumetric quantification of VCAM-MPIO accurately reflected tissue levels of VCAM-1 mRNA; and (3) VCAM-MPIO bound rapidly to target with active sequestration of unbound MPIO in the liver and spleen.

## Introduction

Ischemia-reperfusion injury (IRI) is an important pathological process in acute vascular syndromes including myocardial infarction,[1], [2], [3] stroke,[4], [5] cardiac surgery[2], [6] and organ transplantation.[7], [8] A key feature of IRI is activation of inflammatory pathways, including the endothelial upregulation of adhesion molecules that mediate leukocyte slowing, rolling, and firm adhesion to the vessel wall.[9], [10], [11], [12], [13] Since these adhesion molecules persist on the vascular endothelial surface even after ischemia itself has resolved, their identification could represent a functional imprint or ‘memory’ of the prior ischemic insult.[14] Clinical decision making in acute vascular syndromes is currently hampered by an inability to define the extent and distribution of ischemia. The ability to identify such an imprint non-invasively with magnetic resonance imaging (MRI) could provide more precise and rapid diagnosis and, potentially, guide targeted interventions.

VCAM-1 and its ligand, α_4_β_1_ integrin (also called very late antigen-4, VLA-4), are important mediators of leukocyte recruitment and inflammation, including in IRI.[10] We have recently developed a targeted contrast agent for magnetic resonance molecular imaging. This agent, comprising antibody-conjugated microparticles of iron oxide (MPIO), has shown upregulation of VCAM-1 in a mouse model of cerebral inflammation, artificially induced by direct injection of interleukin-1.[15]

However, if molecular imaging techniques are to be used for diagnosis and monitoring response to therapy, it will be important to establish (1) the sensitivity of detection in more ‘physiological’ conditions and (2) that quantitative contrast effects faithfully reflect tissue levels of the target molecule. The highly uniform size and composition of MPIO provides a ‘quantal’ platform for molecular imaging, whereby the extent of contrast effects might directly report molecular expression within a given tissue. Since VCAM-1 expression is regulated at the level of transcription,[16], [17], [18], [19], [20] we used quantitative real time PCR to test the extent to which objective 3D-quantification of VCAM-MPIO binding reflected tissue levels of VCAM mRNA.

In this study, we (1) investigate the ability of targeted-MPIO to detect VCAM-1 expression non-invasively *in-vivo* and to define its 3-dimensional distribution in a mouse model of unilateral renal IRI; (2) test whether objective automated volumetric quantification of MPIO accumulation, detected by MRI, reflects VCAM-1 messenger RNA expression, measured using quantitative real time polymerase chain reaction (PCR) and (3) define the early time course of both specific VCAM-1 MPIO binding to target and clearance (by the liver and spleen) in order to determine the optimal imaging window.

## Materials and Methods

### Antibody conjugation to microparticles of iron oxide (MPIO)

Purified monoclonal rat anti-mouse antibodies to VCAM-1 (clone M/K2, Cambridge Bioscience) or control IgG-1 (clone Lo-DNP-1, Serotec) were conjugated to myOne tosylactivated MPIO (1 µm diameter; iron content 26%) with p-toluenesulphonyl (tosyl)-reactive surface groups (Invitrogen) according to our previously established method.[15]

### Mouse experimental protocol

This study was undertaken with the approval of the University of Oxford Clinical Medicine Ethical Review Committee and procedures were performed in accordance with the UK Home Office Animals (Scientific Procedures) Act 1986. Male C57BL6/J (H2^b^) mice (12–16 weeks; Charles River) were anesthetized and following an abdominal incision, each renal pedicle was bluntly dissected. Ischemia was induced by clamping the left renal pedicle using a haemostatic microvascular clamp (B-1A, ASSI Corp) for 30 min, while the contra-lateral pedicle was exposed but not instrumented. Cessation of renal blood flow and subsequent reperfusion were confirmed by tissue pallor during occlusion and prompt, uniform return of tissue color after clamp removal.[21] After 16–18 hr reperfusion, mice were subjected to *in-vivo* MRI. All mice received intravenous tail vein injection of either VCAM-MPIO (n = 5) or isotype control IgG-MPIO (n = 3) (4.5 mg iron per kg body weight for both). To determine specificity of VCAM-MPIO, a further group of mice (n = 2) was injected with 0.2 mg of VCAM-1 antibody per kg body weight to block VCAM-1 binding sites with subsequent administration of VCAM-MPIO 15 min later. A control group of mice (n = 2), which underwent no surgical procedure, was also imaged after VCAM-MPIO injection.

### In-vivo MRI

*In-vivo* MRI was carried out on a 9.4-Tesla horizontal magnet interfaced to a VNMRS DirectDrive MR system (Varian Inc. USA) using a quadrature-driven birdcage coil (id 33 mm – Rapid Biomedical, Rimpar, Germany). Mice, anaesthetised with isofluorane (1.5–1.8% isofluorane in 100% O_2_ – flow 3 l/min), were placed prone in dedicated animal cradles. Body temperature was maintained at 37°C using a warm air-blanket. ECG and respiration were monitored continuously throughout the experiment. After scouting, shimming and pulse-calibration, all mice were subjected to a baseline scan prior to MPIO administration using a double-gated (with steady-state maintenance[22]), segmented 3D GE-sequence, optimized to provide bright-blood and T_2_^*^-contrast (TE/TR  = 2.5/4.2 ms, 8 k-space lines per cardiac cycle, TR_seg_ = 1 RR-interval ∼120–170 ms, FOV = 25.6×25.6×22 mm, 18 mm axial slice, flip angle 15°, matrix size 256×256×96, 1 average). The 3D-GE was repeated at six time points, covering a total period of ∼90 minutes post contrast injection.

### MRI Analysis

The external border of each kidney on T2*-weighted images was masked manually prior to segmentation of ‘low signal’ and ‘high signal’ areas of the image, using ImagePro Plus (version 6.1, Media Cybernetics, UK). Low signal (MPIO contrast) and high signal (rapidly flowing blood) areas were defined to be 4 standard deviations either side of the mean signal intensity of the pre-contrast kidney for each animal. These parameters were applied in fully automated fashion to segment each 72-slice sequence that spanned entire kidneys. Segmented images were reconstructed using the 3D Constructor plug-in to visualise the spatial distribution of MPIO binding, with low-signal areas assigned to the green channel and high signal areas to the red channel. The resultant voxel volumes were summated and expressed in µm^3^.

For serial imaging experiments, signal to noise ratios were calculated for peripheral organs using automated signal intensity histograms to quantify the mean signal within each organ of interest. Noise was calculated using the standard deviation of the mean background noise.

### Histology and immunohistochemistry

Following MRI, mice were terminally anesthetised using isofluorane and perfusion fixed using 10 ml PBS followed by 10 ml 4% paraformaldehyde via the left ventricle. Kidneys were cut longitudinally in halves to be snap frozen in liquid nitrogen (for RNA extraction and VCAM-1 immunocytochemistry) or fixed in paraformaldehyde and paraffin embedded (for histology). Paraffin sections of the clamped and unclamped kidney (7 µm thick) were stained with haematoxylin and eosin and examined for the presence of MPIO using light microscopy (100 X objective). Frozen sections were incubated with rat monoclonal antibody to mouse VCAM-1 (Cambridge Bioscience) (1∶50 dilution) overnight at 4°C and detected using a fluorescent anti-rat Alexa Fluor 488 antibody (Invitrogen). Fluorescence images were captured using a Zeiss LSM510 laser-scanning confocal microscope (Zeiss). For immunohistochemistry, liver and spleen sections were stained with primary rat anti-mouse MAC-3 (BD Pharmingen, 1∶100) and biotinylated polyclonal secondary antibodies (Vector Laboratories, Burlingame, California) detected using a peroxidase kit (Vector Laboratories) and 3,3′-diaminobenzidine chromogen (DAB) (Vector Laboratories). Sections were counter-stained with haematoxylin. Digital light microscopy (LM) images of stained sections were captured with a Cool Snap Pro color video camera (Media Cybernetics) mounted on a light microscope (Leica) (x40 and x100 magnification) using ImagePro Plus image analysis software (version 4.5.1; Media Cybernetics).

### Quantitative, real time, reverse transcription polymerase chain reaction (qRT-PCR)

Total RNA was extracted from snap-frozen kidneys using the TRIzol method (Invitrogen) and further purified with an RNeasy Mini kit (Qiagen, Crawley, UK), according to each manufacturer's protocol. Equivalent amounts of purified total RNA were used in 20 µL reverse transcription reactions using a QuantiTect Reverse Transcription Kit (Qiagen) according to manufacturer's protocol. Quantitative Real-time PCR was performed in a StepOne Plus Real-Time PCR System using TaqMan gene expression assays for VCAM-1 and GAPDH with TaqMan Gene Expression Mastermix (Applied Biosystems, Warrington, UK) Data were generated by the comparative threshold cycle (_ΔΔ_ *C_T_*) method by normalizing to Glyceraldehyde 3-phosphate dehydrogenase (GAPDH). StepOne Software version 2.0 (Applied Biosystems) results were exported to Microsoft Excel for further analysis.

### *In-vitro* sEND-1 cell culture

Mouse endothelioma (sEND-1) cells (8×10^5^ per 35 mm well) were treated with 0–10 ng/mL murine-recombinant tumor necrosis factor alpha (TNF-α, R&D systems, UK) for 20 hours at 37°C to induce endothelial VCAM-1 expression. Stimulated cells were incubated in duplicate with VCAM-1-MPIO or negative control isotype IgG-1 MPIO for 30 minutes at room temperature with constant rocking. MPIO binding to cells was assessed by differential interference contrast imaging using a Zeiss LSM510 laser-scanning confocal microscope (Zeiss, Welwyn Garden City, UK).

### Western immunoblotting

Cellular proteins were extracted using RIPA Buffer (Santa Cruz Biotechnology, Santa Cruz, CA) and protease inhibitors (Complete Protease Inhibitor Cocktail Tablets, Roche, Burgess Hill, UK). Proteins were separated by SDS-PAGE, transferred to polyvinylidene fluoride membranes and incubated at 4°C overnight with monoclonal rat anti-mouse VCAM-1 antibody (R&D Systems). After washing, membranes were incubated with an anti-rat secondary antibody (Vector Laboratories, Peterborough, UK) for 60 min at room temperature. Following extensive washing, VCAM-1 protein expression was detected using ECL Western Blot detection reagents (GE Healthcare, Amersham, UK), recorded on X-ray film and quantified by densitometry using ImagePro Plus Software (Media Cybernetics, Silver Spring, MD).

### Statistical Methods

Data are expressed as mean ± SEM and compared by two-tailed Student's *t-*tests. Statistical significance was assigned at *P*<0.05. Linear regression analysis was performed using GraphPad Prism 4 (GraphPad Software Inc, La Jolla, USA) to relate cell-bound MPIO quantification with the dose of TNF-α and for comparing VCAM-1 mRNA expression in the kidneys with MPIO contrast volume.

## Results

### Serial imaging of *in vivo* VCAM-MPIO binding and clearance

**Figure 1:**
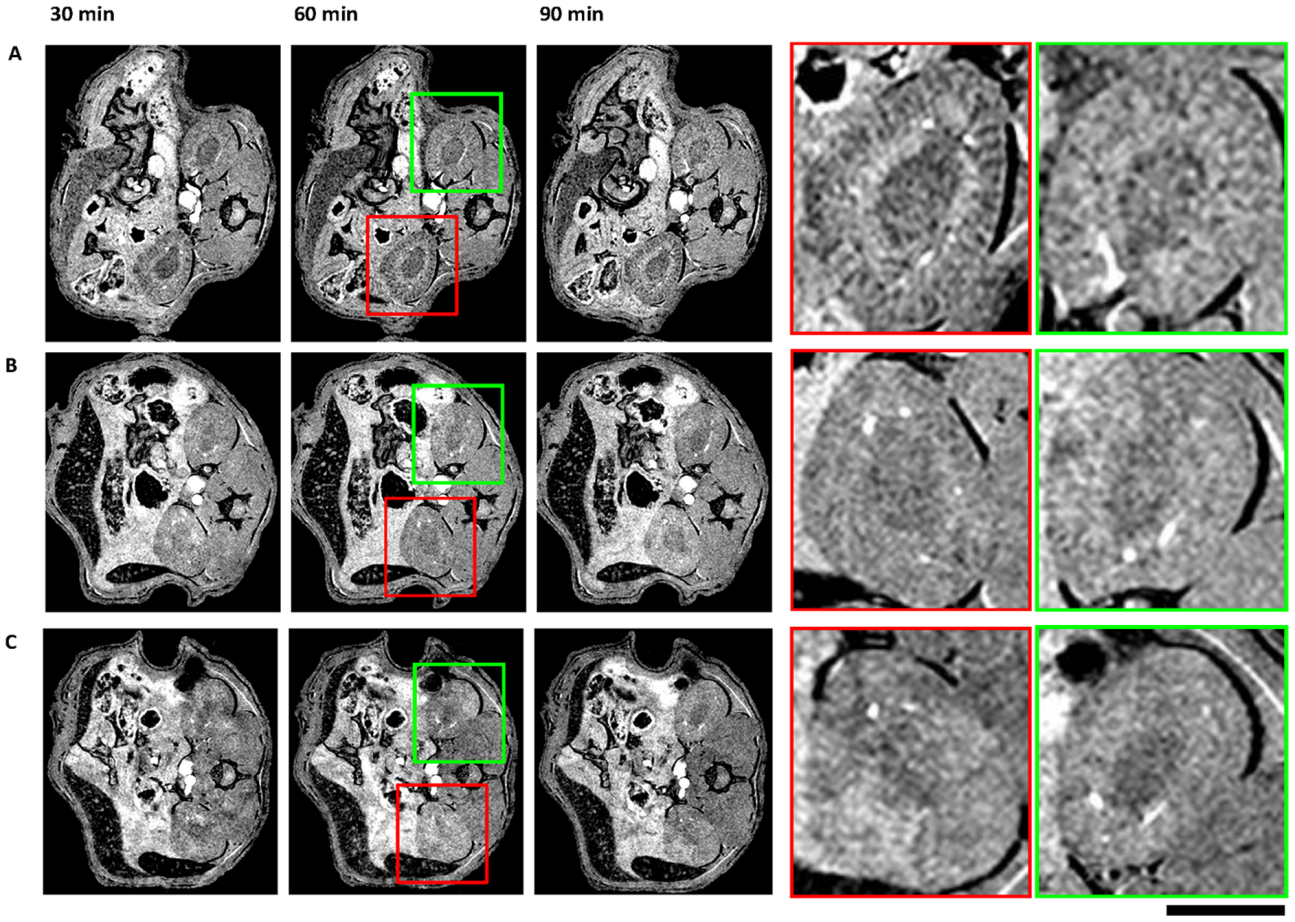
Serial *in-vivo* MRI at 30, 60 and 90 minutes detects VCAM-MPIO accumulation in renal ischemia-reperfusion injury (IRI). **A**. VCAM-MPIO caused a marked contrast effect evident as low-signal in the renal cortex and medulla of the IRI kidney (red box). A lesser degree of signal loss was also evident in the contra-lateral, sham-operated kidney (green box). **B**. Irrelevant isotype IgG-MPIO control showed no contrast effect in either IRI (red box) or sham-operated kidneys (green box) (n = 3). **C**. Pre-treatment of mice with anti-VCAM-1 antibody abolished retention of VCAM-MPIO in both IRI (red box) and sham-operated (green box) kidneys. In A–C, contrast effects peaked at 60 minutes and were sustained throughout the imaging protocol. Scale bar  = 5 mm.

**Figure 2:**
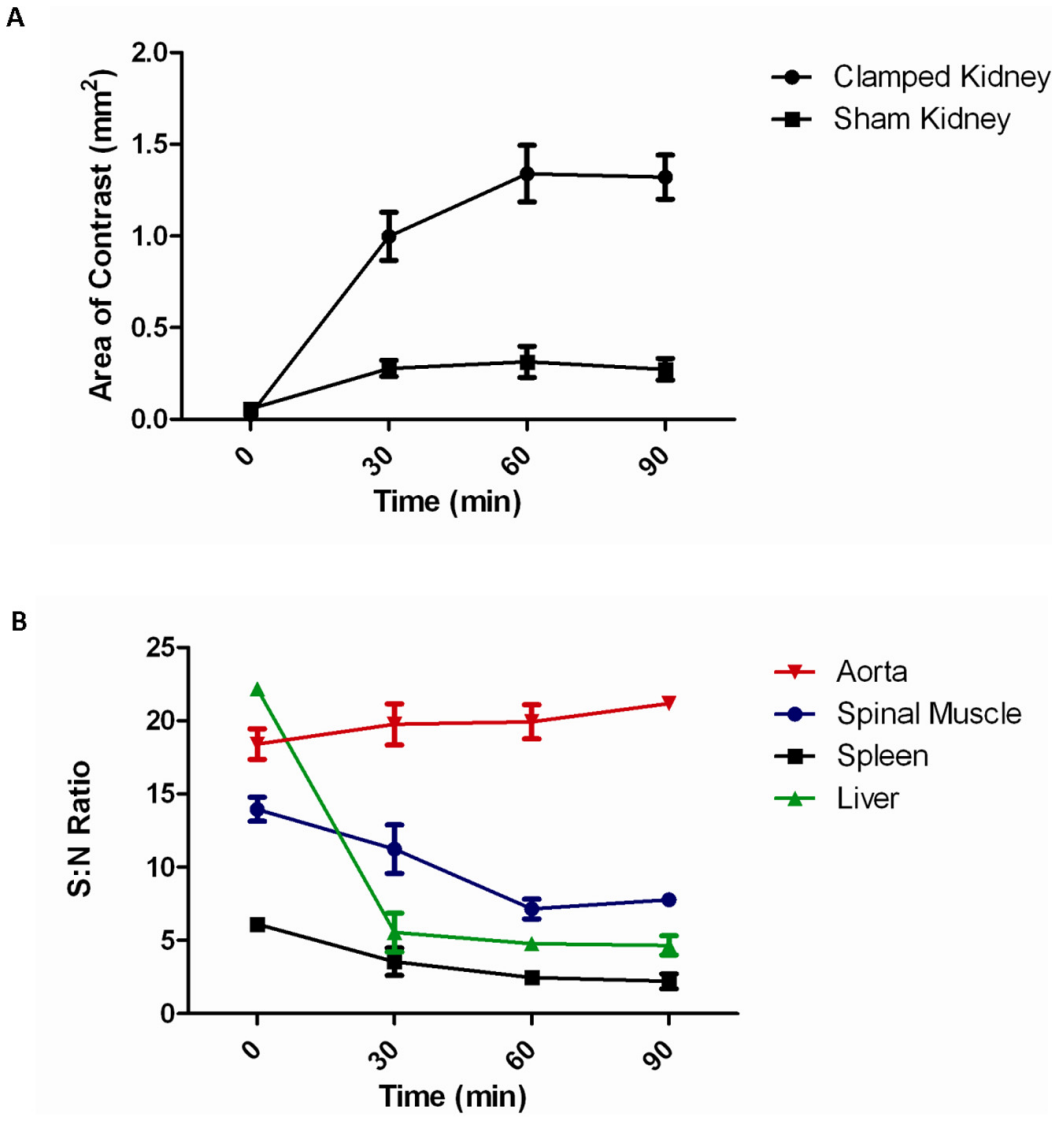
Signal to noise ratios and area of contrast effect as functions of time. **A**. Contrast uptake of VCAM-MPIO (low signal areas) was evident in both clamped & sham operated kidneys by 30 minutes, and was maximal by 60 minutes. Contrast was stable from 60 to 90 minutes post contrast-injection. **B**. Signal to noise ratio (SNR) was calculated for intra-abdominal organs. SNR in the liver fell steeply within the first 30 minutes reaching steady state by 60 min post-contrast administration.

In kidneys subjected to IRI, VCAM-MPIO caused a marked contrast effect that was evident as areas of low-signal intensity in both the renal cortex and medulla. VCAM-MPIO binding was rapid, showing contrast effects within 30 minutes, and persisted for the entire 90 minute imaging period (Figure 1). To determine the optimal time point for imaging, we undertook serial MRI at 30 minute intervals, demonstrating that contrast effects were clearly apparent by 30 minutes and maximal by 60 minutes with persistent specific contrast in the kidneys at 90 minutes (Figure 2A). Microparticles of iron oxide were rapidly cleared by the liver and spleen, as indicated by reduction in signal to noise ratio in those organs within 30 minutes (Figure 2B).

### Automated segmentation and quantification of contrast volume

**Figure 3:**
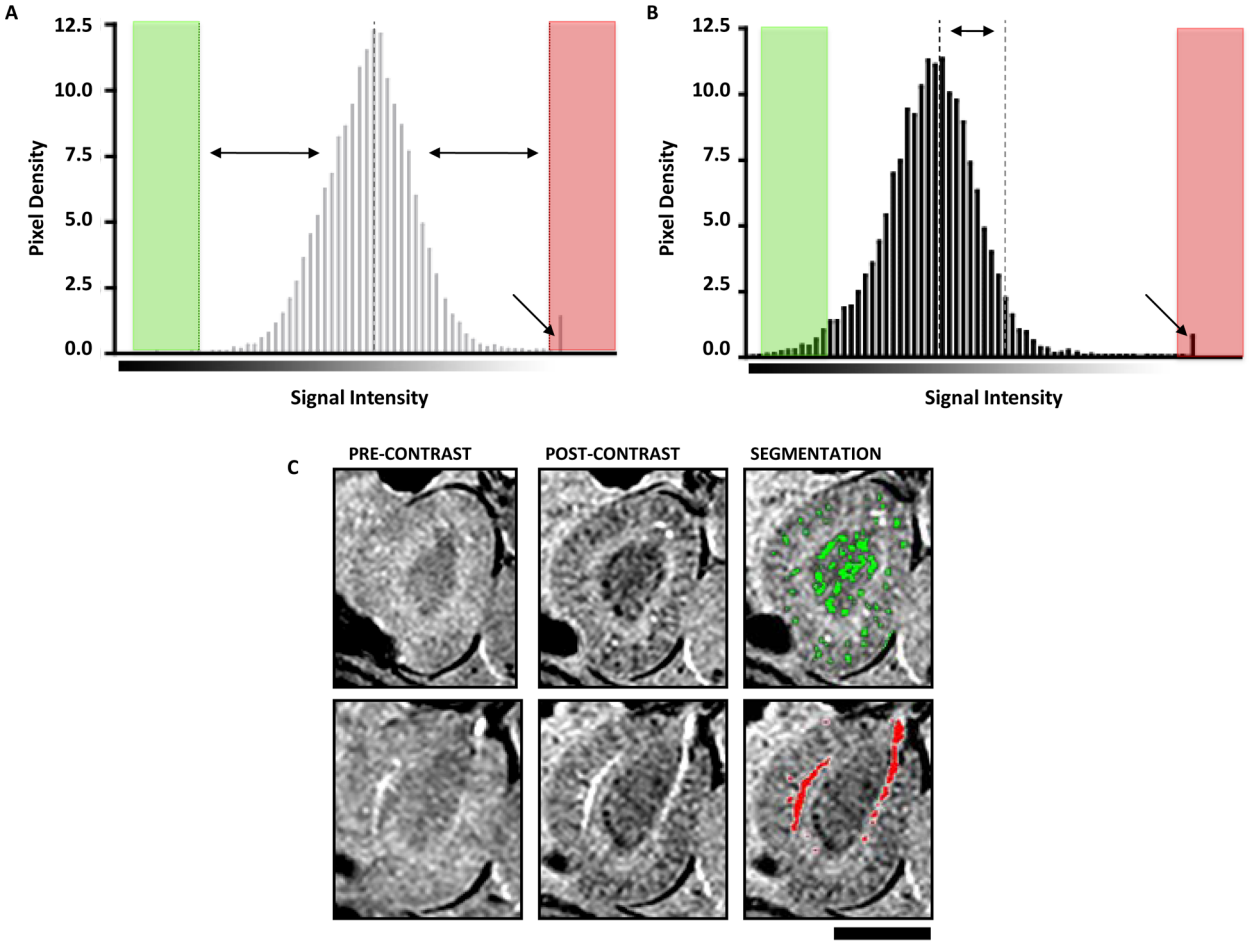
Automated segmentation and quantification of MPIO accumulation. **A**. Pre-contrast signal intensity histogram showing mean (dashed line) and 4 standard deviations from the mean into the black spectrum (green) and 4 standard deviations from the mean into the white spectrum (red). The arrow indicates a peak in the white spectrum corresponding to the intensely bright signal from the renal artery. **B**. Signal intensity histogram 60 minutes post-contrast shows a leftward shift in the mean towards the black spectrum (dashed line) as a result of the presence of MPIO in the kidney. In this post-contrast spectrum, dark pixels 4 standard deviations from the original mean were designated to show contrast effects for the purposes of segmentation. The bright signal from flowing blood in the renal artery is unaltered by MPIO (arrow). **C**. Automated histogram-based segmentation of MPIO contrast effects (green, top panel) and the renal artery (red, bottom panel) from the same slice. Segmentation enabled 3D volumetric quantification and reconstruction.

In order to define the volumetric distribution of MPIO contrast and obtain a ‘bright blood’ arteriogram, using objective criteria, we examined signal intensity histograms pre-contrast (Figure 3A) and 60 minutes post-contrast (Figure 3B). To ensure stringent segmentation criteria, we imposed a threshold four standard deviations from the mean for both low signal (iron-based contrast) and high signal (bright blood). The ability of this signal intensity algorithm to provide automated segmentation of *both* iron related contrast effects (green) *and* the renal arterial structures (red) from the same image was tested (Figure 3C).

### Quantitative volumetric analysis of binding of microparticles of iron oxide

**Figure 4:**
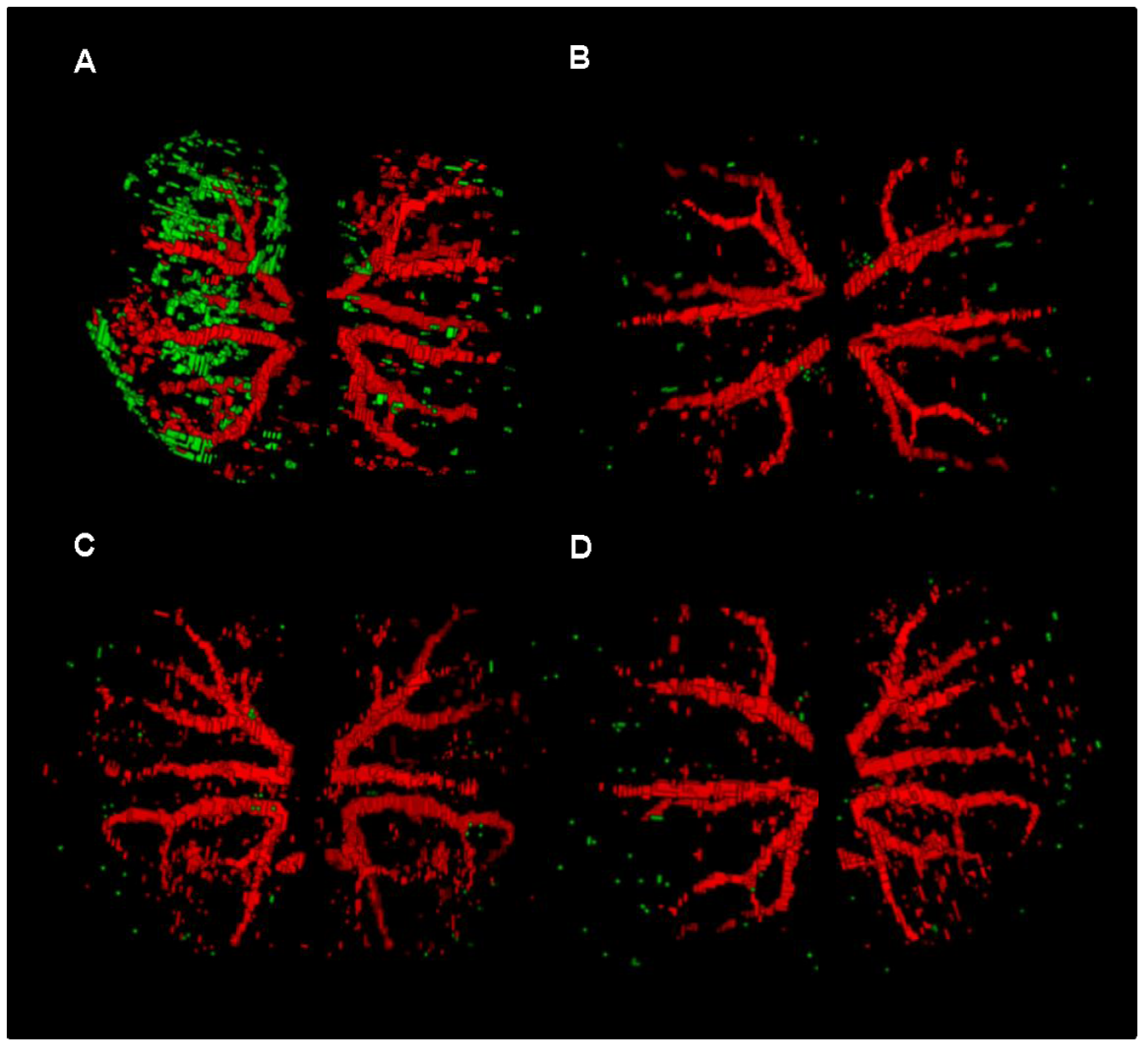
3D reconstruction of segmented kidneys. **A**. VCAM-MPIO contrast (green) was abundant and in both the medulla and cortex of IRI kidneys (image-left) and to a lesser extent in sham-operated kidneys (image-right). **B**. In mice that underwent an identical protocol, there was little to no binding of the isotype IgG-MPIO in either kidney. **C.** Similarly, there was little to no VCAM-MPIO retention in either kidney in mice that underwent no surgery (n = 2) or **D**. in mice pre-treated with anti-VCAM-1 antibody prior to contrast administration.

**Figure 5:**
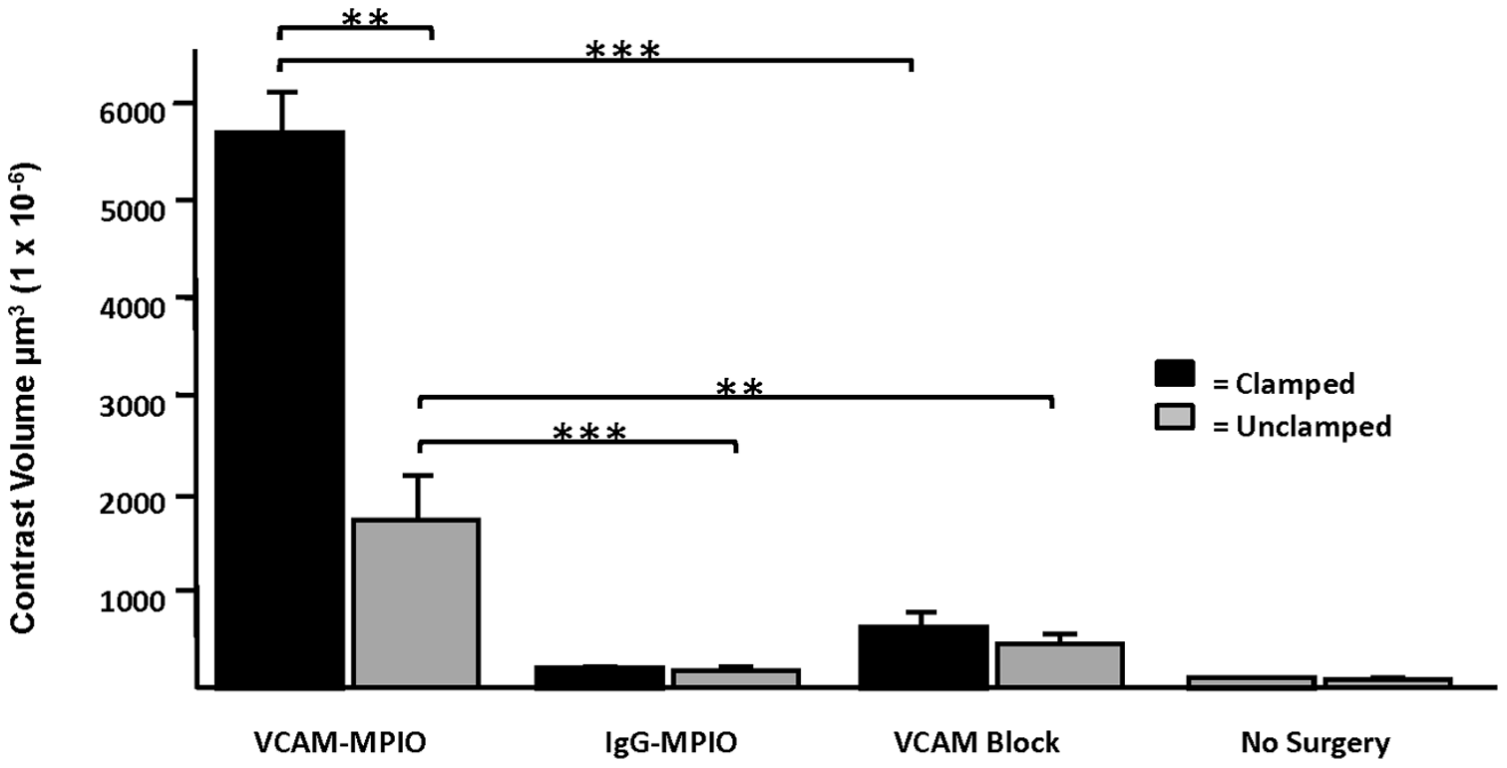
Quantitative volumetric analysis of MPIO binding. VCAM-MPIO related contrast effects were significantly upregulated in clamped versus unclamped kidneys (n = 5, ** *P*<0.01). Blocking with anti-VCAM-1 antibody resulted in significantly less contrast volume (n = 2, ***P<0.001). Unclamped kidneys showed significant contrast volume compared to both sham operated kidneys in animals given non-specific isotype IgG-MPIO (n = 3, ***P<0.001) as well as animals given anti-VCAM-1 prior to VCAM-MPIO administration (n = 2, **P<0.01).

Three-dimensional (3D) volumetric maps of contrast were constructed from the segmented images and related to renal anatomy, defined by the branching arterial tree (Figure 4). VCAM-MPIO retention was highly conspicuous in the cortex and medulla of kidneys that were subject to IRI (Figure 4A, image-left kidney). There was little or no binding of isotype control IgG-MPIO in either the IRI or sham operated kidneys (Figure 4B). Pre-treatment of mice with VCAM-1 antibody prevented VCAM-MPIO retention in both kidneys (Figure 4C)**.** Similarly, mice that underwent no surgery and were injected with VCAM-MPIO showed very little MPIO binding (Figure 4D). There was sparse, but definite, binding of MPIO in sham operated kidneys (Figure 4A, image-right kidney). Kidneys subject to IRI (5991±354×10^6^ µm^3^) showed a 69-fold increase in VCAM-MPIO contrast compared to kidneys with no surgical intervention (87±7×10^6^ µm^3^, *P*<0.001) (Figure 5). Contra-lateral sham operated kidneys showed modest, but definite contrast effects (1740±528×10^6^ µm^3^, *P*<0.01) while pre-treatment with VCAM-1 antibody effectively blocked VCAM-MPIO accumulation in IRI kidneys (625±80×10^6^ µm^3^, *P*<0.001). Virtually no contrast effect was seen in either clamped or sham operated kidneys exposed to IgG-MPIO (90±8×10^6^ µm^3^ and 112±7×10^6^ µm^3^, respectively).

### Quantitative RT-PCR analysis of VCAM-1 mRNA expression

**Figure 6:**
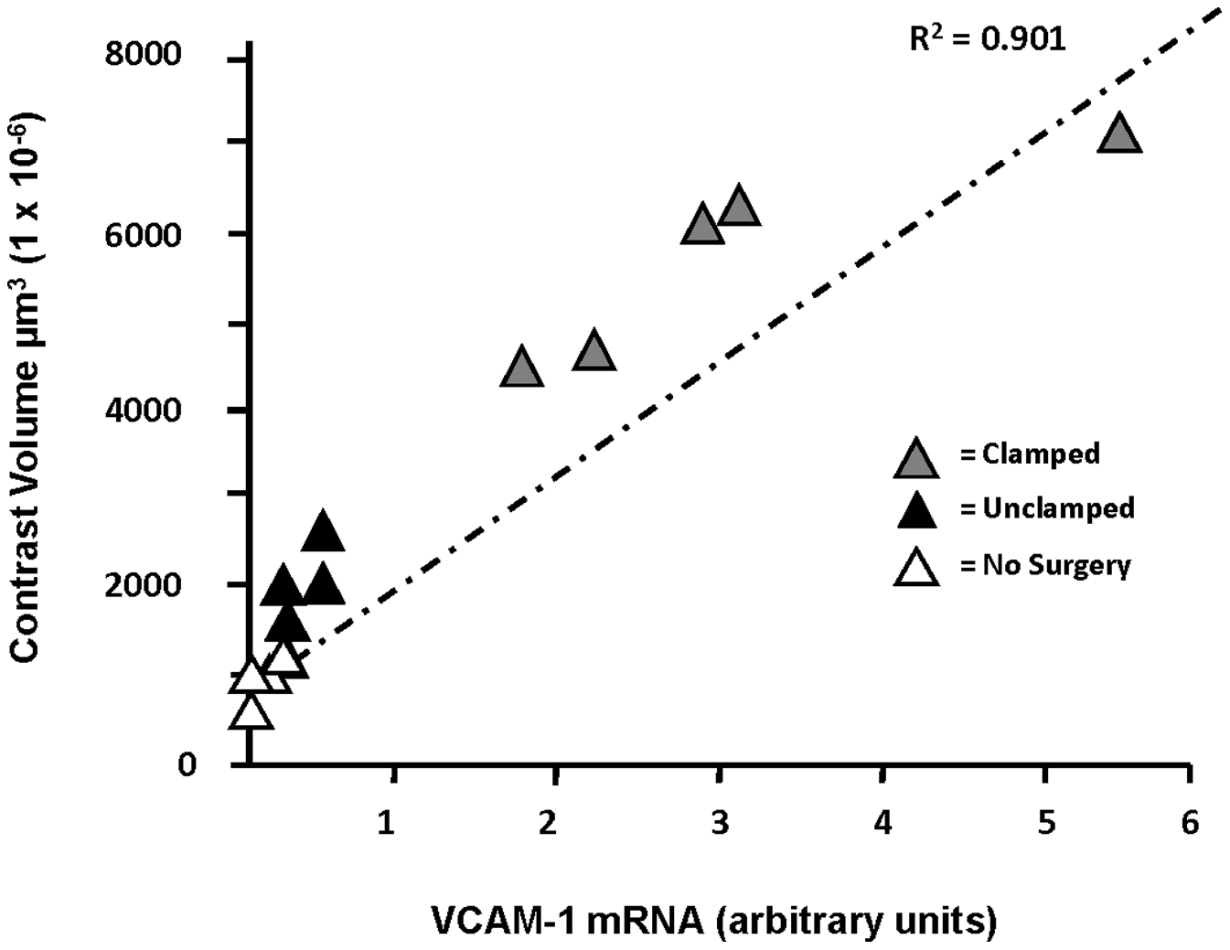
MPIO-contrast volume reflects VCAM-1 mRNA expression in tissue. VCAM-1 mRNA expression was 12-fold higher in clamped kidneys versus sham operated kidneys (n = 5, *P*<0.01). VCAM-1 mRNA expression in sham operated kidneys was significantly upregulated compared to no surgery animals (*P*<0.05). VCAM-1 mRNA expression and VCAM-MPIO related contrast volume was highly correlated (R^2^ = 0.901).

To test whether quantitative VCAM-MPIO contrast measurements reflected tissue levels of target, VCAM-1 mRNA expression in kidneys was measured using qRT-PCR. VCAM-1 expression was 12-fold higher in clamped kidneys compared to sham operated kidneys (3.06±0.63 *vs.* 0.25±0.08, *P*<0.01) and 65-fold higher than kidneys without surgical intervention (0.05±0.02, P<0.001). VCAM-1 mRNA expression in sham-operated kidneys was significantly upregulated compared to kidneys with no surgical intervention (*P*<0.05) (Figure 5). VCAM-1 mRNA expression and VCAM-MPIO contrast volume were highly correlated (R^2^ = 0.901, P<0.01) (Figure 6).

### Distribution of VCAM-1 and microparticles of iron oxide on histology

**Figure 7:**
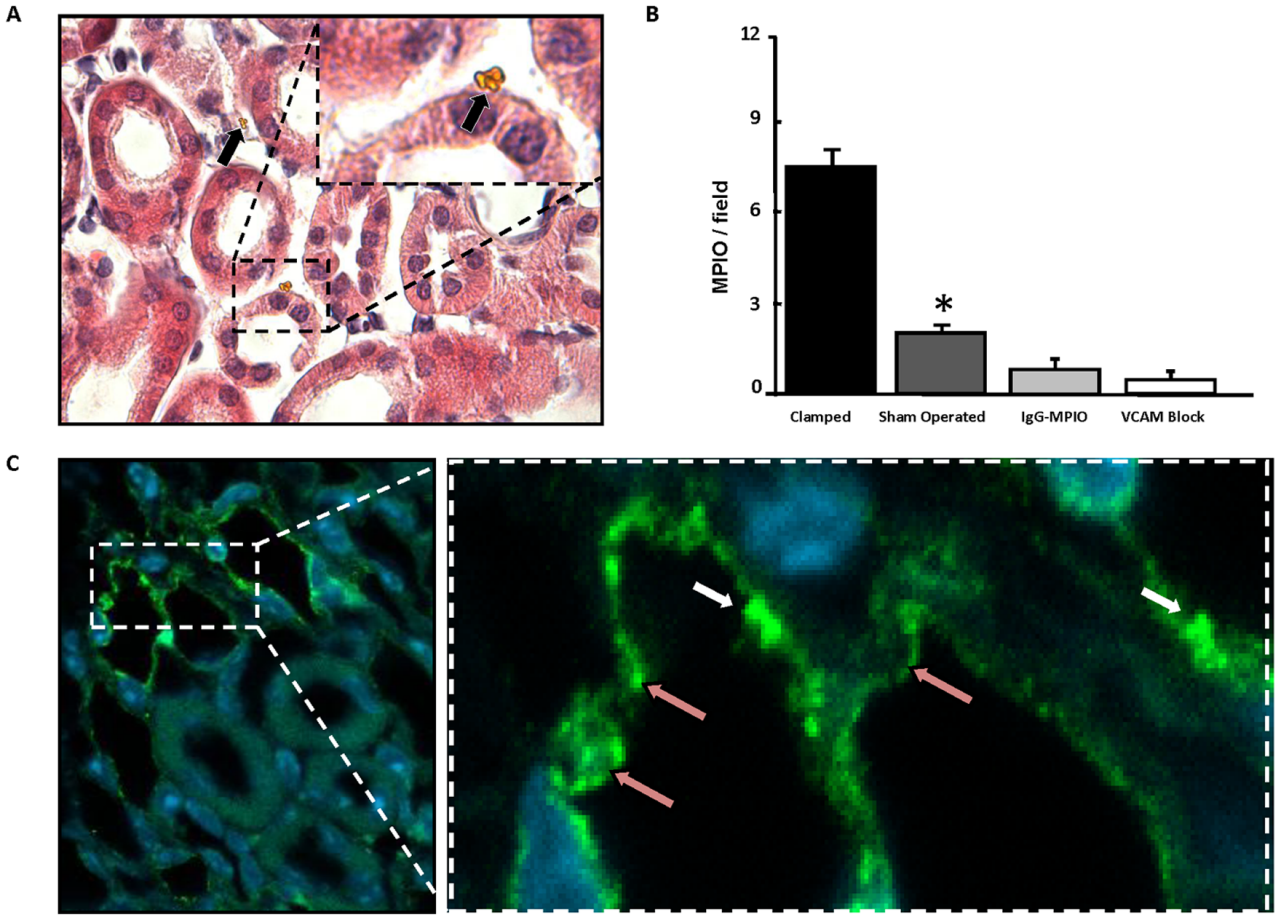
Histology. **A**, Haematoxylin & eosin stained section of IRI kidney with VCAM-MPIO confined to vascular spaces in peritubular capillaries (black arrows). A small cluster of MPIO is present (Inset) **B.** VCAM-MPIO retention was significantly upregulated in clamped kidneys versus sham operated kidneys (n = 2, *P<0.05) (6 fields of view per kidney, magnification: 100X) **C**, VCAM-1 immunofluorescence revealing VCAM-1 expression in vessels, but not in tubular cells. VCAM-MPIO bearing the same antibody that was used as the primary for VCAM-1 immunofluorescence (pink arrows) appear as intensely bright spheres (white arrows).

On microscopic inspection of IRI kidneys, VCAM-MPIO were adherent to the vessel wall, either singly or in small clusters, of the peritubular capillaries (Figure 7A). MPIO retention was not associated with local infarction or haemorrhage. Quantification of MPIO under light microscopy (Figure 7B) confirmed the quantitative relationships determined by *in vivo* MRI. VCAM-1 immunofluorescence was confined to vascular structures without expression in the renal tubules and in association with VCAM-1 MPIO retention (Figure 7C).

**Figure 8:**
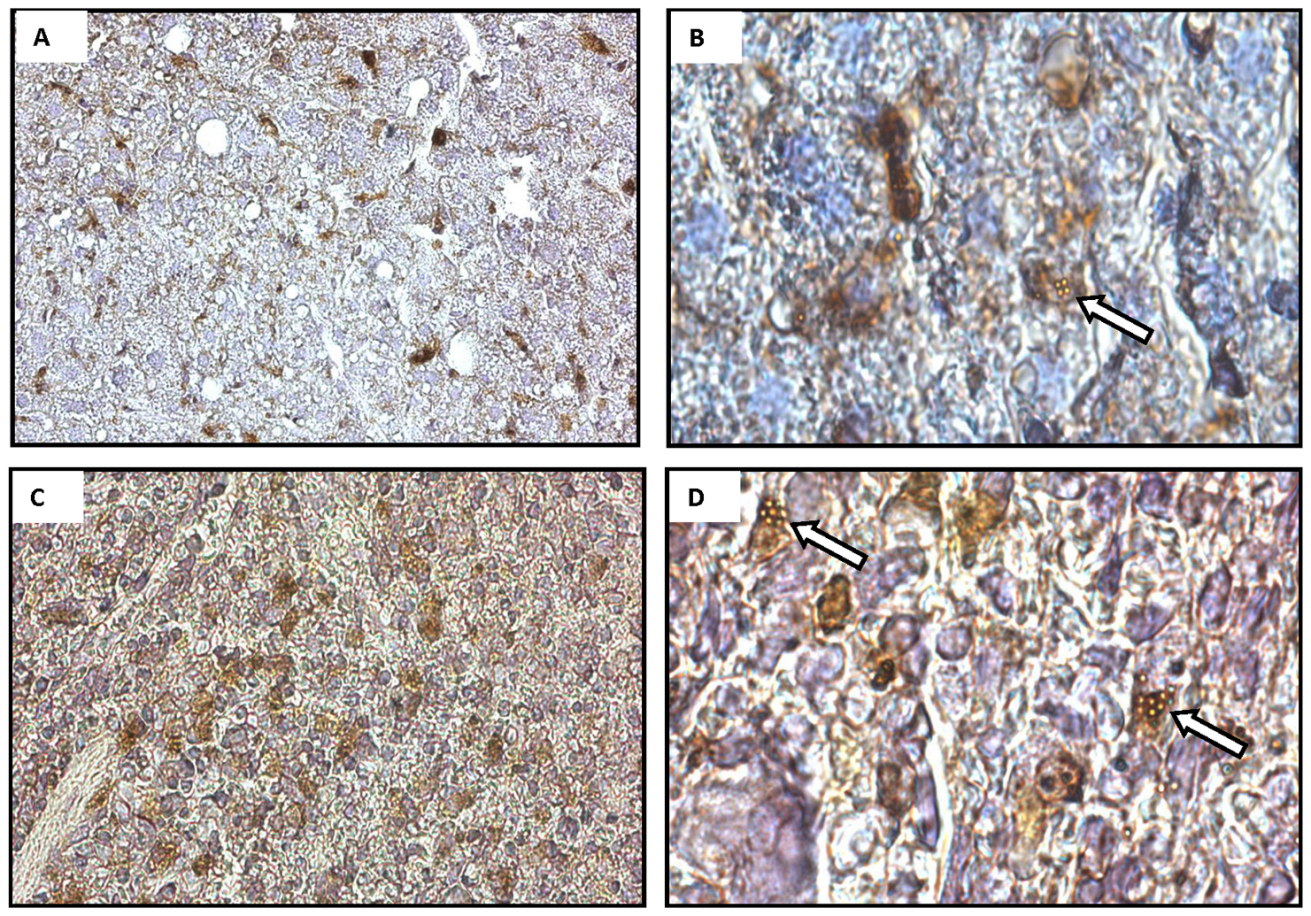
Representative images of MAC-3 immunostaining for macrophages. (A) liver and (B) spleen (haematoxylin counterstain, magnification x 40). Panels C and D show co-localisation of MPIO within cells that stained positively for MAC-3 in (C) liver and (D) spleen (magnification x 100).

In order to demonstrate that the IRI in this model induced cellular inflammation, kidneys from additional mice were immunostained for Gr-1, demonstrating neutrophil accumulation 24 hours after injury in the IRI mice, but not in the uninjured control (Figure S1). Unbound MPIO were sequestered in cells that stained positively for MAC-3 in both liver (peri-sinusoidal, Kupffer cells; Figures 8A & B) and spleen (monocyte/macrophages; Figures 8C & D).

## Discussion

We provide evidence for a non-invasive *in-vivo* molecular magnetic resonance imaging approach for quantification of VCAM-1, a key mediator of inflammation. Applied in a mouse model, this technique enabled the identification of ischemia-reperfusion injury in kidneys and described the 3-dimensional anatomical distribution of inflammation in relation to the renal arterial tree. We report an objective segmentation technique for automated quantification of the contrast effect and confirm that this faithfully reflects tissue levels of VCAM-1 transcript using real time quantitative real time RT-PCR. This approach therefore reveals ‘ischemic memory’ that is of potentially broader diagnostic utility in the depiction of a persisting physiological consequence of recent ischemia. In acute vascular syndromes, such a tool, to define ‘volume at risk’, could identify high-risk patients and inform on response to treatment, including in the evaluation of new therapies.

### Quantitative imaging

The diagnostic utility of molecular imaging is enhanced if the contrast effects reflect tissue levels of target, since such quantitative imaging would potentially allow severity of disease to be determined and response to treatment monitored. VCAM-1 has been imaged previously using MPIO;[15] nanoparticles of iron oxide;[23] and acoustic microbubbles[24], [25] Although the contrast effects can be quantified, these earlier studies did not investigate whether there was a quantitative relationship between non-invasive contrast measurements and tissue levels of target. Micron-size range iron oxide-based molecular imaging probes provide both greatly enhanced sensitivity due to their high iron content and, because of their uniform size and composition, an opportunity for quantitative evaluation. Since VCAM-1 expression is regulated at the level of transcription,[16], [17], [18], [19], [20] we used quantitative real time RT-PCR to measure tissue levels of VCAM-1 mRNA in the kidneys, and demonstrated a close correlation between *in vivo* VCAM-MPIO retention and *ex vivo* measurement of VCAM-1 mRNA. To provide further confirmation of the quantitative link from mRNA to MPIO binding, we stimulated sEND-1 cells with graded doses of TNF-α, showing strong correlations between mRNA, protein and MPIO retention (Figure S2). Therefore, MRI can be used to attain quantitative reporting that is usually only achievable with nuclear techniques, e.g. positron emission tomography.[26]

### Sensitivity and Dynamic Range

Renal ischemia is known to cause inflammation at sites distant to the ischemic tissue and has been implicated in injury to the liver,[27] lungs[28], brain[29] and heart.[30] The mechanisms are not fully understood, but mediators may include interleukin-6, since the pulmonary inflammatory response was attenuated in IL-6 knockout mice.[28] In the model used here, the high degree of sensitivity provided by MPIO enabled the detection of inflammation (confirmed with real time RT-PCR), remotely in the contra-lateral kidney, which had not been exposed to ischemic injury. This intermediate inflammatory level was detectable with VCAM-MPIO allowing demonstration of both the sensitivity and dynamic range of this molecular imaging technique.

### Molecular imaging platform

The MPIO contrast agent provides a platform approach to molecular MRI that allows the substitution of alternative ligands with specificity for a range of endovascular targets.[31] We have previously demonstrated the potential to target VCAM-1 or P-selectin in isolation and have identified synergistic effects of dual-targeted MPIO for binding under conditions of high shear.[32] Van Kasteren *et al* have generated saccharide-based synthetic ligands that bind with high affinity to selectins, and which have been applied to molecular MRI in the brain.[33] Indeed, P-selectin is an attractive target for acute inflammation imaging, including “ischemic memory”, since the synthesized protein is stored within the Weibel-Palade bodies of endothelial cells and can be expressed on the cell-surface within minutes by transportation to the plasma membrane.

### Microparticles of iron oxide: kinetics and distribution

High ‘target to background’ contrast benefits from rapid onset of binding at target combined with swift clearance from the blood pool. Unlike contrast agents that need to be concentrated within cells or to permeate tissue beyond the vasculature, MPIO binding to the endothelium is rapid, occurring within minutes. Furthermore binding is directly dependent on its interaction with a highly accessible target without intervening processes (e.g. cell membrane transporter function, vessel permeability, tissue diffusion) that have the potential to confound quantitative reporting of the molecular target. Rapid specific accumulation at sites of inflammation (within 30–60 minutes) combined with clearance to the liver (within 30 minutes) recommended the 60 minute time point as optimal for determination of contrast effects in this model. Therefore the size and physical properties of MPIO confer both contrast sensitivity *and* favourable binding and clearance kinetics.

### Alternative approaches for MR contrast

The molecular imaging approach described here targets molecules expressed on activated vascular endothelium. Ligand-conjugated MPIO are designed to mimic the binding of peripheral blood leukocytes, but are not dependent on leukocyte binding *per se*. As a consequence, binding at a site of target up-regulation can be rapid, a property that would be valuable in the context of a clinical contrast agent. An alternative approach has been to use cells loaded with MPIO as Trojan horses and to utilise MRI to follow their accumulation according to the trafficking and distribution of these cells. This has been applied to track stem cells[34], [35], [36] and macrophages.[37] The time course of accumulation of such cells is much longer than for the ligand-conjugated MPIO, so that they are potentially better suited tracking chronic processes and regenerative therapies than the acute inflammatory process exemplified by IRI.

### Limitations

Short-term ill effects of MPIO were not seen in mice. We have previously reported the bio-distribution of 1.0 µm diameter MPIO, showing a lack of retention in the kidneys and lungs at both 30 minutes and 24 hours.[38] The MPIO used here were non-biodegradable and are not suitable for use in humans. However, iron-oxide containing contrast media are already in clinical use and it should be feasible to synthesize biodegradable particles.[39], [40] Before clinical use is contemplated, it will clearly be necessary to undertake a full toxicological evaluation, including the analysis of MPIO effects on liver and kidney function as well as the potential of antibody-conjugated MPIO to elicit an immune response in the recipient. The last possibility could be ameliorated by the use of ‘humanised’ antibodies.

### Conclusions

VCAM-MPIO detected VCAM-1 expression and defined its 3-dimensional distribution, revealing ‘ischemic memory’ in mouse kidneys. Furthermore, automated volumetric quantification of MPIO accurately reflected tissue levels of VCAM-1 mRNA across a biologically relevant dynamic range, providing evidence for quantitative molecular magnetic resonance imaging.

## Supporting Information

Figure S1 VCAM-1 molecular imaging in this study was intended to reveal expression of the pro-inflammatory mediator VCAM-1. In order to demonstrate that the degree of ischemia-reperfusion injury (IRI) would eventually lead to a cellular inflammatory response, kidneys subject to 30 minutes ischemia and 24 hours reperfusion were compared to unoperated controls. A, non-ischemic kidney without inflammatory cell infiltration. B, immunostaining (brown) for Gr-1, demonstrating neutrophil infiltration by 24 hours.
(1.39 MB TIF)

Figure S2 Increased VCAM-MPIO retention reflects VCAM-1 mRNA and protein levels *in vitro*. A, VCAM-MPIO retention increased in a dose dependent manner in sEND-1 cells following TNF-α stimulation for 24 hours *in vitro* (n = 4/group, R2  = 0.88). B, VCAM-1 mRNA expression was 6-fold higher in sEND-1 cells treated with 10 ng/ml TNF-α (black bar) versus cells treated with 2 ng/ml (gray bar, **P<0.01) and 14-fold higher than unstimulated cells (white bar, ***P<0.001). C, VCAM-1 protein, assessed semi-quantitatively, was 2-fold higher in sEND-1 cells treated with 10 ng/ml TNF-α (black bar) versus cells treated with 2 ng/ml (gray bar, *P<0.01). VCAM-1 expression was 7-fold higher in cells treated with 2 ng/ml TNF-α versus unstimulated cells (white bar, *P<0.01).
(0.24 MB TIF)
