# Supplementary figures and images for: In Vivo Quantification of Vcam-1 Expression in Renal Ischemia Reperfusion Injury Using Non-Invasive Magnetic Resonance Molecular Imaging
Source: PLoS One. 2010 Sep 21;5(9):e12800. doi: 10.1371/journal.pone.0012800 (PMC2943468; doi:10.1371/journal.pone.0012800)

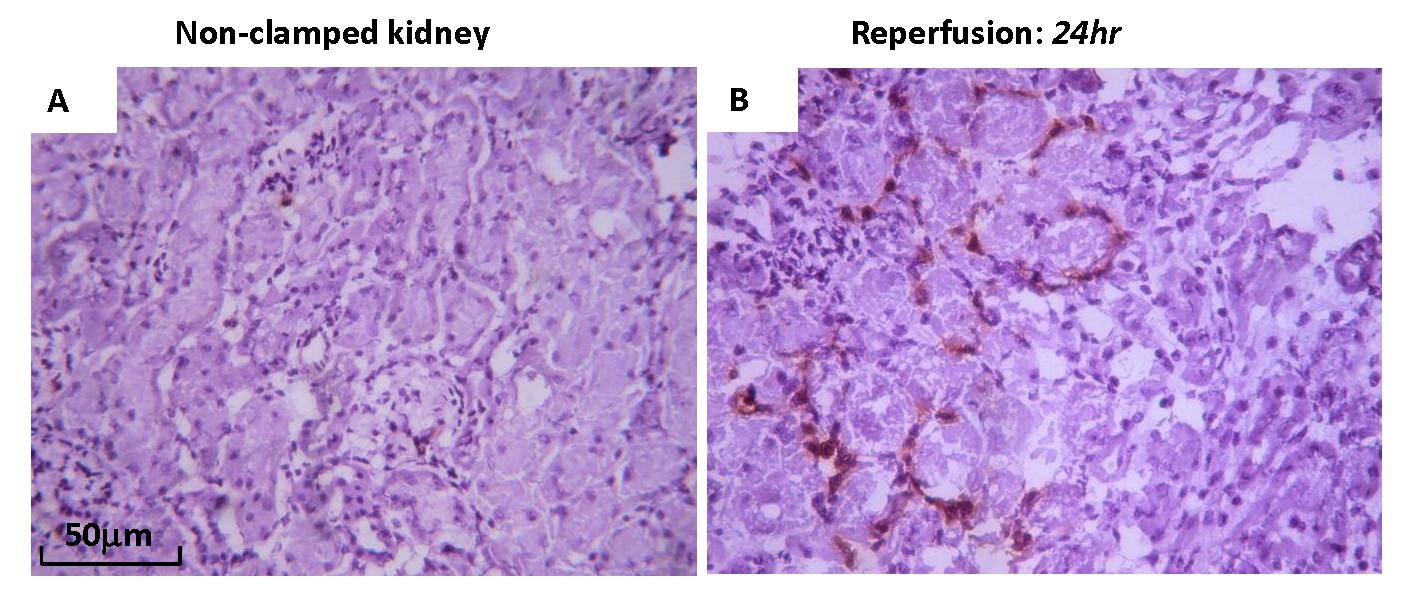

Supplement: Figure S1 — VCAM-1 molecular imaging in this study was intended to reveal expression of the pro-inflammatory mediator VCAM-1. In order to demonstrate that the degree of ischemia-reperfusion injury (IRI) would eventually lead to a cellular inflammatory response, kidneys subject to 30 minutes ischemia and 24 hours reperfusion were compared to unoperated controls. A, non-ischemic kidney without inflammatory cell infiltration. B, immunostaining (brown) for Gr-1, demonstrating neutrophil infiltration by 24 hours. (1.39 MB TIF) [file pone.0012800.s001.tif]

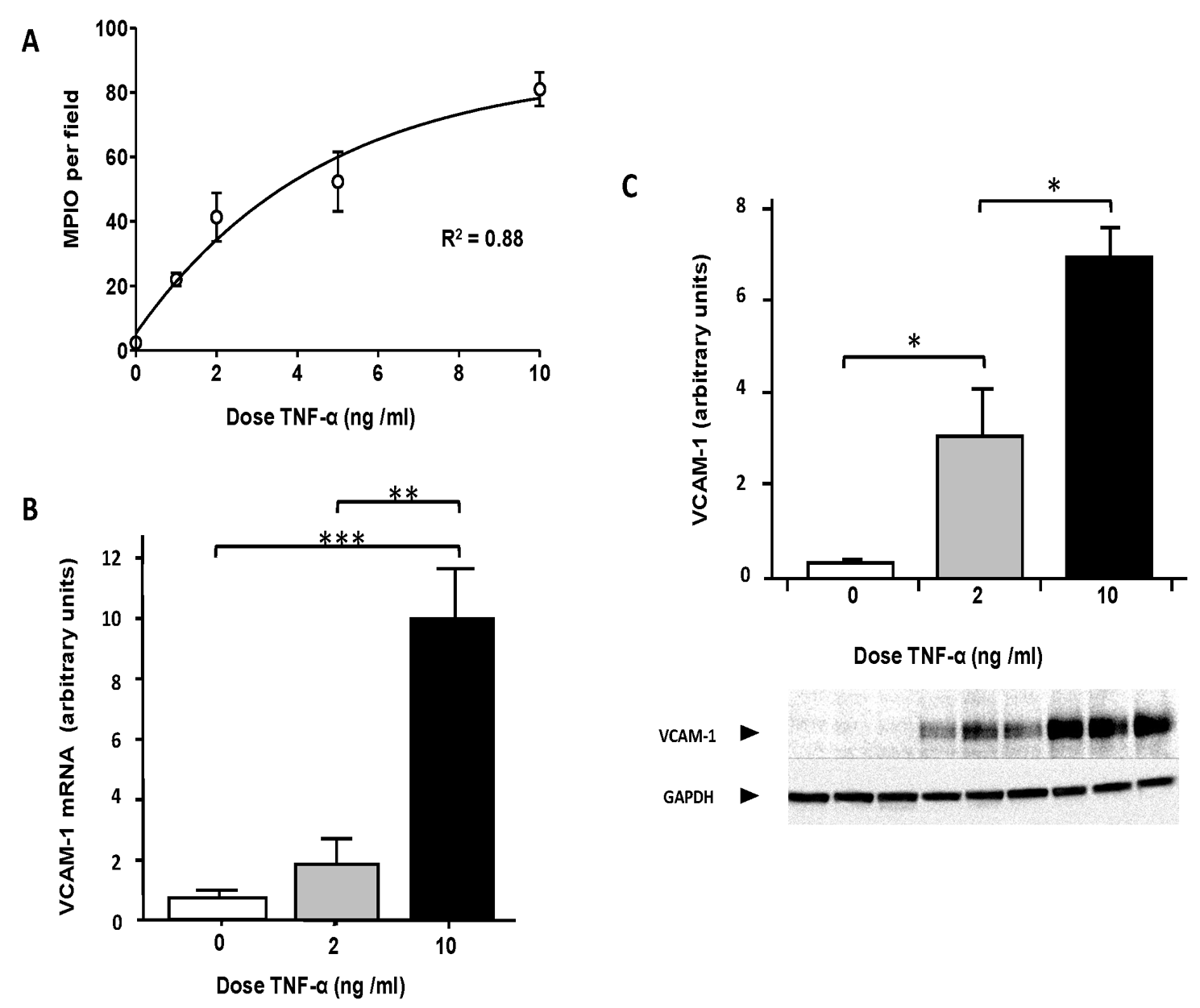

Supplement: Figure S2 — Increased VCAM-MPIO retention reflects VCAM-1 mRNA and protein levels in vitro. A, VCAM-MPIO retention increased in a dose dependent manner in sEND-1 cells following TNF-α stimulation for 24 hours in vitro (n = 4/group, R2 = 0.88). B, VCAM-1 mRNA expression was 6-fold higher in sEND-1 cells treated with 10 ng/ml TNF-α (black bar) versus cells treated with 2 ng/ml (gray bar, **P<0.01) and 14-fold higher than unstimulated cells (white bar, ***P<0.001). C, VCAM-1 protein, assessed semi-quantitatively, was 2-fold higher in sEND-1 cells treated with 10 ng/ml TNF-α (black bar) versus cells treated with 2 ng/ml (gray bar, *P<0.01). VCAM-1 expression was 7-fold higher in cells treated with 2 ng/ml TNF-α versus unstimulated cells (white bar, *P<0.01). (0.24 MB TIF) [file pone.0012800.s002.tif]
